# Leveraging Patient/Community Partnerships to Disseminate Patient Centered Outcomes Research in Geriatrics

**DOI:** 10.3390/geriatrics4020035

**Published:** 2019-05-11

**Authors:** Lee A. Lindquist, Anne Seltzer, Chris Forcucci, Norine Wong, Vanessa Ramirez-Zohfeld

**Affiliations:** 1 Division of General Internal Medicine and Geriatrics, Department of Medicine, Northwestern University Feinberg School of Medicine, Chicago, IL 60611, USA; aseltzer@nm.org (A.S.); Vanessa-ramirez-0@northwestern.edu (V.R.-Z.); 2 Aging and In-Home Services of Northeast Indiana, Fort Wayne, IN 46805, USA; cforcucci@agingihs.org; 3 First Vitals Health and Wellness, Inc., Honolulu, HI 96813, USA; norine.wong@firstvitals.com

**Keywords:** dissemination, community partners, geriatrics, patient-centered outcomes research

## Abstract

**Background:** With the growth of patient-centered outcomes research (PCOR), partnerships between researchers and patients have presented novel opportunities for disseminating results. A large gap exists in disseminating patient-centered research results to older adult patient end-users. We sought to examine the experiences of patient/community partners in disseminating PCOR results and characterize lessons learned that may facilitate future researcher-patient/community dissemination partnerships. **Methods:** Patient/community partners who participated in geriatrics-focused PCORI-funded research and were active in disseminating results, as part of their planned activities, were recruited for one-to-one qualitative interviews. Constant comparative and thematic analysis were used to identify and describe common themes that emerged in a survey of open-ended questions. **Results:** Ten individuals (four community partners, six patients) aged 55–87 years were interviewed. Analysis revealed that for successful dissemination, subjects felt it was vital to reach out to people affected by the results, leverage personal stories, and tap into pre-developed programming. Patient/Community partners identified potential audiences through word-of-mouth, community requests, and mapping a list of audiences—targeting those directly affected as well as those who worked with the audience (e.g., not specifically medical). Patient/community partners recommended that researchers engage patient/community partners for suggestions on audiences, show empathy, include diverse populations, and maintain a community-focus. One community partner stated, “Why wouldn’t we help disseminate results? It’s a no-brainer. We know people!” **Conclusion:** Patient/community partners provide effective ways to communicate results, new audiences to reach, improved communication with different audiences, and improved credibility of the findings. The lessons learned have implications for assisting future research-patient/community partnerships in the dissemination of their patient-centered research.

## 1. Introduction

As medical research evolves to become more patient-centered [1,2,3,4], patient and community partnerships with researchers have provided increasing opportunities for engagement and community-based participatory research [5]. The establishment of the Patient-Centered Outcomes Research Institute (PCORI) in 2010 to fund comparative effectiveness research (CER) boosted engagement of patients and community partners as research partners as a new way of pursuing clinical research. Being a contract requirement, all PCORI-funded projects engage patients and community groups as partners in the multiple stages of research. Patient partners are defined as patients who represent the study population of interest, as well as their family members, and caregivers that represent them; community partners are defined as non-patient individuals who represent the interests of the study population based on professional, rather than personal, experience, and include clinicians, healthcare purchasers, industry representatives, hospital/health system administrators, and policy makers [6]. From an analysis of PCORI-funded projects, each project had between one to seven patient or community partners (mean age 54 yrs.), with the majority being female (70%), white (78%), and most commonly represented the patient/consumer (29%), caregiver (12%), or clinician (15%) communities. Almost 90 percent of PCORI awardees engage patients and clinicians as partners, according to annual progress reports from awardees. Increasingly, patients, caregivers, clinicians, and other community partners have shown interest in research roles that emphasize usefulness and understandability of research [7]. Prior work has shown that the most common phase of engagement between patients and researchers has been outcome and measurement identification (75%) [8]. The current evidence base is extremely limited in leveraging patients and community partners for dissemination and implementation of patient-centered research results [9].

A vital part of research is disseminating findings to potential users of the results. Dissemination has been defined as the targeted distribution of information and intervention materials to a specific clinical practice audience and implementation as the use of strategies to adopt and integrate evidence-based health interventions and change practice patterns within specific clinical settings [10]. In a study of 260 annual reports of PCORI-funded studies, patient partners were engaged for dissemination in 43% of the studies (n = 101). Investigators and partners reported partner involvement in dissemination activities as co-presenting at scientific meetings, developing manuscripts, determining avenues to share findings, writing newsletters, participating in media interviews, and speaking with public health officials about the project. Unfortunately, dissemination does not always reach the people who would most likely benefit from the results. Clinicians, patients, and caregivers do not often experience effective dissemination and have indicated unmet needs for incorporating research findings into clinical decision-making. Less than half of patients and caregivers reported exposure to any type of comparative effectiveness findings [11]. When research especially does not reach the hands of older adults, there are potential detriments to their health. Older adults experience more multi-morbidity and increased healthcare needs when compared to younger cohorts. Subsequently, older adults would expect to benefit the most from up to date research findings and yet, they are unlikely to experience dissemination of research findings. This lack of awareness of research findings is considerable for older adults. Considering that research has shown that it takes approximately 17 years for 14% of new practice approaches to reach the bedside [12], partnering with patient/community partners may provide a means of expediting the process.

Researchers have recognized the need for additional knowledge in leveraging patient/community partnership opportunities [13]. Most respondents were familiar with (81%) and interested in (87%) partnering with patients and/or caregivers in research. There is a significant opportunity for researchers to engage patients and caregivers as partners, but gaps exist in understanding how to leverage those opportunities in dissemination [14]. In our previous patient centered research, we found a lack of information on how to guide patient/community partners on dissemination. To our knowledge, there are no published “how to disseminate research” available for patient/community partners. Similarly, there was a gap in how to guide researchers in partnering for dissemination of research with patients and community members. With our patient/community partners, we sought to fill this gap and provide written guidance or “how to” for future patient/community partners and researchers to disseminate patient centered research.

## 2. Methods

### 2.1. Participants

All 10 of the patient and community partners from the PCORI-funded studies, Advance Planning for Home Services for Seniors [15,16] and Leveraging Patient Partner/Stakeholder Engagement to Implement PCORI—PlanYourLifespan.org [17] were invited to participate and consented. There were no refusals. In order to be consented and surveyed, participants had to speak English, be at least 21 years old, and have participated as a patient or community partner in one of the aforementioned studies. The study was considered exempt by the Northwestern University Institutional Review Board (IRB).

### 2.2. Interviews

A semi-structured interview guide was utilized for each interview, consisting of open-ended questions and follow-up probes to encourage discussion. All interviews were held confidentially and were conducted by an experienced qualitative interviewer, between May to September 2017, and lasted approximately 60 min. A brief socio-demographic questionnaire was also included.

### 2.3. Analysis

Interviews were audio-recorded and transcribed verbatim. Transcripts were analyzed by two authors (L.L., V.R.Z.) using content and constant comparative techniques [18], through which the coders independently assessed participant responses for focal themes before convening to compare and compile their findings to create a preliminary list of categories and major themes. The coders met multiple times to discuss and refine the identified themes and triangulate their perspectives. Identified discrepancies were resolved through discussion; there were no cases in which the coders were unable to reach consensus. The coders then organized the content into an overarching categorical system. It is common to use multiple coders in the development of such categorical systems to control for the subjective bias each coder brings to the analytic process. From these overarching categories, the coders reached agreement on themes that were particularly relevant. Descriptive statistics were used to analyze the participant surveys.

## 3. Results

Ten adults (all female; 4 community partners, 6 patients) with ages ranging from 55 to 87 (with a mean age of 71.6 years, sd 8.2) completed the interview. There were no refusals among invited participants. All participants had some amount of post-graduate education and reported living in a home, either alone (44.4%) or with a spouse (55.6%). Of the participants, primary roles included patients, caregivers, social workers, area agency on aging nurse leader, and staff members, professional organization leaders, and clinicians.

### 3.1. Overarching Themes Associated with Successful Dissemination

When asked “What are the most important factors for dissemination & implementation of PCOR?”, patient/community partners identified four central reasons:

#### 3.1.1. Reaching out to Those Most Affected by the Results and Their Shared Experiences with the Results

The patient/community partners were chosen for the original PCOR studies as they were representative of the study population. This commonality helped patient/community groups identify the people most affected by the results. A common theme across partners in this cohort was the desire to make it personal when reaching out to those most affected: “Well it’s real important, I’m going through it right now.”

#### 3.1.2. Leveraging Connections to the Study Population (e.g., Parents, Children, Caregivers)

Several participants sought out the opportunity to not necessarily connect with the same age group as those in the PCOR study, but people who were younger and intersecting with the study population:

“And as much as it is for the older adults, we have also found this very helpful for the adult children. Because not only is it two-pronged, one it helps them plan for the relatives that they’re caring for and then for themselves as well.”

#### 3.1.3. Determining the Practical Application of Results

Patients/Community partners were less concerned with the statistical analytics and more concerned with the practical applicability of the intervention:

“Well, first of all, the practical value of it. We come across people all the time, just in my line of work at my agency with families who are so confused. This work helped to clear up the confusion.”

#### 3.1.4. Tapping into Pre-Developed Groups and Programming

As patient/community partners were linked heavily with their own communities, they were able to identify pre-developed groups and programming that would be served by the PCOR results.

“I’ve talked to the family caregivers at my caregiver support group, my pastor at church and my senior friends and I’ve talked to family caregivers who have been referred to me and I’ve talked to other facilitators at support groups.”

“We presented to just a variety of different community groups, we did some presentations at the agency where we just invited folks in, we did some caregiver presentations specifically for caregivers. Obviously we presented to a lot of older adults who they themselves would be interested in the information but also the caregivers who were very interested. And then we also presented to professionals: attorneys, we presented to some bank, like the finance officers, the trust officers, the ones that really work with older adults, so those kinds of groups. And we shared information — so not just presentations — we shared information at a wide variety of places. So you know we shared the information with our home delivery meals folks that don’t necessarily get out to congregate meal sites, we put information at the library, we shared it, we gave handouts to ministers, they shared it from the pulpit. So it got shared very, very widely.”

### 3.2. Practical Steps to Disseminate and Implement PCOR Results

In considering future dissemination of PCOR among patient/community partners, we asked our patient/community partners on the specifics of how they disseminated PCOR results.

#### 3.2.1. Identifying Potential Audiences for Dissemination

Patient/community partners cited that they made a list of audiences, targeting end-users as well as those who worked with end-users. They examined their personal networks and reach and contacted those most likely to benefit. They then used word-of-mouth and many times experienced community group requests for more information. Several partners brought it up naturally, in conversations:

“It came naturally in conversation with people about where they are as they are talking about situations. It seems to be very prevalent now in what they want in their future or what should happen to them next.”

“Yes, I actually disseminate it pretty regularly, I would say a couple of times a week in clinic. I have patients who will say, “How can I better prepare for the future?” Or, “These are my concerns,” and I’ll say, this is a great website that just kind of gives you, leads you towards what you should be thinking about in these five topics.”

#### 3.2.2. Designing Relatable Messages to Present PCOR Results.

We asked our patient/community partners about how they presented the PCOR results and how delivery/messaging changed across audiences. Common themes used were to

(1) Start with a question “Has this experience happened to you or someone you know? Have you done this yet? Have you thought about this at all?”
(2) Be prepared for personal stories and if wanted, use a personal story of the PCOR results.
  “I think you know with a lot of this just be prepared because you are going to get a lot of stories. And so that to me, that feels very comfortable because I listen to a lot of stories in my job as a social worker but for a lot of people this sparks a memory for them or an opinion that they have”
(3) Make the results relatable and tout it as a resource. When possible, demonstrate hands on the PCOR results, encourage use of computers, smart phones if electronic on ascertaining results.
  “My personal belief is being able to actually show people, demonstrate and if possible be able to have a hands-on opportunity for them was definitely more advantageous for people to connect.”

#### 3.2.3. Further Advice to Future Patient/Community Partners

When asked “What advice would you give to other community/patient partners who plan on disseminating PCOR?”, our cohort responded with the following themes: (1) Go through the PCOR results yourself first; familiarize yourself with it and consider what questions you or others might have. (2) Know your audience; Use a sensitive approach as you do not know outright what others prior experiences are with the PCOR findings. (3) Try to make the dissemination/uptake of PCOR results a positive experience. What would be helpful is to make sure that it is a positive experience and that you are aware of all of the positive sides of it for whatever group you are in front of. (4) Look beyond your classic—age and groups can be expanded to widen dissemination and actively pursue options. (5) Repeat the message often in your outreach.

“I think you just have to attack it. I mean, you just, when you reach out to somebody ask, is there anybody else you know or any other organization that you think would be willing to hear this information that I could call on?”

### 3.3. Further Advice to Future PCOR Researchers

We asked our patient/community partners, “What advice would you give to researchers to improve/promote PCOR results?” and received useful information that were characterized into the following themes:

#### 3.3.1. Keep Focus in the Community

“It’s probably self-evident but I guess to me the biggest issue is to stay focused in the community. You know, to make sure that your network is broad and that you, it makes me think that is key.”

#### 3.3.2. Show Empathy to Those Who Will Be Affected by the PCOR Results

“My advice to researchers is to remember that whatever it is you are promoting, that you at some point may have to use it too. And to promote it as though you were on the other side of it, on the receiving end for you or a wonderful, loving family member.”

#### 3.3.3. Reach out to and Consider Messaging to Diverse Populations

“I would say you know just try and reach out to as diverse population as you are able to because we had talked about trying to get (the results) translated into Spanish as well which would be very helpful but I think also learning more about cultural norms and what would actually be helpful based on peoples customs.”

#### 3.3.4. Engage Patients/Community Partners on Suggestions for Who, What, and Where to Present

“Being able to engage PCOR patients/community partners and being able to listen to what they have to say without overriding what you think they should be getting is really important. You’ve got to have patients and community partners involved in planning your research and in operationalizing it.”

## 4. Discussion

Community-based partners and patients can be invaluable in disseminating PCOR results, since they are able to directly reach patients. This research set to characterize the experiences and insights of patient/community partners to assist with future partnerships with researchers in disseminating PCOR results. This is the first study to our knowledge of its kind to directly examine patient/community partner involvement in direct dissemination. While there is a wealth of literature about patient-centered care, outcomes and measurement, dissemination is a critical piece of research. In conducting these qualitative interviews, we found that engaged patients/community partners were able to leverage connections in the community as well as identify innovative routes of dissemination (e.g., non-medical—financial, legal), which health researchers may not consider. As PCORI-funded research requires patient/community partners who have experiences or missions that align with the studies, patient/community partners are able to leverage shared similar experiences with those who would benefit from the results and thus be able to form personal connections in disseminating to the community.

While this study highlights the unique capacity of patient/community partners in disseminating of PCOR results, limitations also presented. As the PCOR results disseminated were mainly focused on older adult issues, it would be interesting to determine if this could be generalized to research involving younger populations. Our patient/community partners were also all female, with post-graduate education, which further limits the generalizability of the study. Further research is needed on the effects with diverse populations as well as a larger cohort. Finally, a real-world follow up of how these dissemination themes impacted communities over the long-term would be a valuable addition to the study design, in order to identify the extent of each dissemination method.

### Implications for Disseminating Patient Centered Outcomes Research

This research is the first to our knowledge that identifies strategies that patient and community partners have used in disseminating PCOR. The lessons learned have unlimited implications for assisting future research-patient/community partnerships in the dissemination of their patient-centered research.
